# Standardization of TSH and FT4 to gestational age in early pregnancy and associations with clinical outcomes

**DOI:** 10.1530/ETJ-24-0344

**Published:** 2025-07-22

**Authors:** Joris A J Osinga, Layal Chaker, Sjoerd van den Berg, Vincent W V Jaddoe, Eric A P Steegers, Henning Tiemeier, Robin P Peeters, Tim Korevaar

**Affiliations:** ^1^ The Generation R Study Group, Erasmus University Medical Center and/or Sophia Children’s Hospital, Rotterdam, The Netherlands; ^2^ Departments of Internal Medicine, Erasmus University Medical Center and/or Sophia Children’s Hospital, Rotterdam, The Netherlands; ^3^ Academic Center For Thyroid Diseases, Erasmus Medical Center, Rotterdam, The Netherlands; ^4^ Departments of Epidemiology, Erasmus University Medical Center and/or Sophia Children’s Hospital, Rotterdam, The Netherlands; ^5^ Departments of Clinical Chemistry, Erasmus University Medical Center and/or Sophia Children’s Hospital, Rotterdam, The Netherlands; ^6^ Departments of Pediatrics, Erasmus University Medical Center and/or Sophia Children’s Hospital, Rotterdam, The Netherlands; ^7^ Departments of Obstetrics and Gynecology, Erasmus University Medical Center and/or Sophia Children’s Hospital, Rotterdam, The Netherlands; ^8^ Departments of Child and Adolescent Psychiatry, Erasmus University Medical Center and/or Sophia Children’s Hospital, Rotterdam, The Netherlands

**Keywords:** thyroid, pregnancy, FT4, TSH, gestational age

## Abstract

**Background:**

To account for pregnancy-specific changes in thyroid physiology, international guidelines recommend the use of trimester-specific reference intervals. However, the pragmatic division in trimesters does not necessarily align with the changes in thyroid physiology. While the goal of treating gestational thyroid dysfunction is to prevent thyroid hormone-mediated adverse events, it remains unclear which method of standardizing to gestational age, if any, is most effective in identifying individuals at higher risk of adverse pregnancy events.

**Methods:**

We included 5,675 women participating in a population-based prospective cohort with data on thyroid-stimulating hormone (TSH), free thyroxine (FT4) and thyroperoxidase antibodies (TPOAbs) during early pregnancy (median: 13.2 weeks, 95% range: 9.8–17.6). We studied the association of TSH and FT4 with pre-eclampsia, premature delivery, birth weight and offspring IQ with or without full gestational age standardization of TSH and FT4 using multivariable regression models.

**Results:**

There was a positive association of gestational age at blood sampling with TSH (difference in mean TSH: +9.6%; *P* < 0.001) and a negative association with FT4 (difference in mean FT4: −20.2%; *P* < 0.001). Standardizing TSH to gestational age led to reclassification of 36 women as having normal TSH (9.9%) and 27 as having abnormal TSH (0.5%). For FT4, 62 women were reclassified as having normal FT4 (20.3%) and 57 as having abnormal FT4 (1.1%). Standardization of TSH and FT4 concentrations led to an attenuation of the associations with any outcome of up to 71% as compared to non-standardized TSH or FT4.

**Conclusions:**

Full standardization of TSH and FT4 to gestational age either does not affect or weakens their associations with clinical outcomes, suggesting that accounting for gestational age offers no benefit with regard to identifying high-risk thyroid dysfunction during early pregnancy.

## Introduction

Adequate maternal thyroid hormone production is required during pregnancy for maintaining metabolic homeostasis and ensuring sufficient transplacental passage of thyroid hormone to the fetus. Thyroid physiology changes considerably during pregnancy due to human chorionic gonadotropin (hCG) production, an increase in thyroid hormone-binding proteins, placental type 3 deiodinase expression and the placental transfer of thyroxine (1, 2). To account for gestational changes in thyroid-stimulating hormone (TSH) and free thyroxine (FT4), international guidelines advocate the use of trimester-specific reference intervals for maternal TSH and FT4 (3, 4). However, the pragmatic division in trimesters (i.e., an arbitrary cut-off at 13 weeks, when hCG levels peak) may not be the optimal approach for establishing reference intervals during gestation.

The importance of gestational thyroid dysfunction is often illustrated through the association with adverse pregnancy outcomes and offspring development (3, 4, 5). Consequently, trials assessing levothyroxine treatment strategies typically focus on the incidence of adverse outcomes and/or measures of offspring neurodevelopment (6, 7, 8, 9). One potential reason for the lack of consistent treatment effects in these trials is a suboptimal definition of thyroid dysfunction, which may dilute treatment effects by failing to accurately target the subgroup of women at highest risk. It is therefore essential to optimally define gestational thyroid dysfunction as a benchmark for future trials.

Accounting for gestational age for the assessment of thyroid function tests has been done for over two decades (10), based on seminal studies in the 1990s, which described physiological changes in thyroid hormones in pregnancy (1, 11, 12). International guidelines currently recommend accounting for these changes using center and trimester-specific reference intervals (3, 4, 13). However, approaches for adjusting to gestational age are rarely compared in terms of their risk of clinical outcomes, which limits the evidence supporting trimester-specific reference intervals and gestational age adjustment overall.

Arguments against the division in trimesters include that trimesters do not align with the natural physiology of thyroid changes in pregnancy, as the most relevant shifts in TSH and FT4 occur around the transition from the first to second trimester (1). In addition, previous research has found minimal differences in the reference intervals between the first and second trimesters, particularly for TSH (14), suggesting that trimester-specific reference intervals offer limited added value in accounting for physiological changes. Since the ultimate goal of detecting thyroid dysfunction in pregnancy is to prevent adverse pregnancy outcomes, the optimal strategy to identify which of the abovementioned two options is superior is to assess the risk of adverse pregnancy outcomes, which have previously been associated with gestational thyroid function (5, 15, 16, 17, 18), as assessed using both methods.

In order to investigate the added value of standardizing reference intervals to gestational age in pregnancy (i.e., trimester specific or any other standardization), we aimed to study the association of gestational TSH and FT4 concentrations, with and without gestational age standardization with adverse clinical outcomes. In addition, given that hCG is the main determinant of early pregnancy changes in TSH and FT4 concentrations, we additionally analyzed TSH and FT4 concentrations standardized to hCG.

## Methods

### Design and population

This study was embedded in Generation R, a population-based prospective cohort from early fetal life onward in Rotterdam, the Netherlands (19). All women with gestational TSH, FT4, hCG and/or thyroperoxidase antibodies (TPOAbs) measurements available were eligible. Women with multiple pregnancy, pre-existing thyroid disease, using thyroid-interfering medication or that had undergone fertility treatment were excluded. Thyroid function tests were determined in stored serum after pregnancy, thus preventing treatment based on the thyroid function tests measured in the context of this cohort. The general design, all research aims and the specific measurements in the Generation R Study have been approved by the Medical Ethical Committee of the Erasmus Medical Center, Rotterdam. Written informed consent was obtained from all participants.

In order to study the difference in the association of non-standardized versus standardized TSH and FT4 with adverse clinical outcomes, we revisited previously studied datasets, in which we investigated the association of maternal thyroid function with pre-eclampsia (standardized TSH/FT4 available in 5,153 out of 5,153 originally included subjects), premature delivery and birth weight (standardized TSH/FT4 available in 5,662 out of 5,971 originally included subjects) and offspring IQ (standardized TSH/FT4 available in 3,649 out of 3,839 originally included subjects) (20, 21). In a separate analysis, as a proof of concept and analysis of consistency, we standardized TSH and FT4 to hCG concentrations based on the fact that hCG is a major determinant of gestational age-related changes in TSH and FT4 concentrations (1).

### Serum measurements

Maternal serum samples were obtained in early pregnancy (median 13.2 weeks, 95% range 9.8–17.6). Serum tubes were centrifuged and serum was stored at −80°C. TSH and FT4 were determined in maternal serum samples using chemiluminescence assays (Vitros ECi; Ortho Clinical Diagnostics, USA; TSH Cat# 191 2997, RRID:AB_3101993; FT4 Ortho Clinical Diagnostics Cat# 138 7000, RRID:AB_3101994). FT4 values were corrected (+23%), as compared to the manufacturer recommended calibration, to calibrate to the reference measurement procedure of equilibrium dialysis (22). Similarly, TSH values were corrected (−11%) to calibrate to the reference measurement procedure used in the clinic (Siemens Immulite XP2000i; (23)). The intra- and interassay coefficients of variation were <4.1% for TSH and <5.4% for FT4 (24). Maternal total hCG concentrations were analyzed in same serum using an Immulite XPi system (Siemens Healthcare Diagnostics, USA), details of which have been described previously (25).

### Clinical outcomes

The definition of all outcomes used for this study has previously been described in detail (20, 21, 26, 27, 28). In short, certified medical doctors reviewed women’s hospital charts and defined pre-eclampsia according to the criteria of the International Society for the Study of Hypertension in Pregnancy. Pre-eclampsia was identified as the development of a systolic BP of 140 mmHg or greater and/or a diastolic BP of 90 mmHg or greater (at least two BP readings) after 20 weeks of gestation in a previously normotensive woman, plus the presence of proteinuria (defined as two or more dipstick readings of 2 or greater, one catheter sample reading of 1 or greater, or a 24 h urine collection containing at least 300 mg of protein). Information on birth weight and gestational age at birth was obtained from community midwives, obstetricians and hospital registries. Birth weight was assessed using standard deviations scores, standardized to gestational age at birth, and the scores were constructed using the Niklasson percentile growth curves. Premature delivery was defined as a gestational age at birth <37 weeks or <34 weeks (very premature delivery). Non-verbal child IQ was assessed using two subtests of a validated Dutch nonverbal intelligence test, the ‘Snijders-Oomen niet-verbale intelligentie test’ (median age of 6 years (95% range 5.6–7.9 years). This test broadly assesses the spectrum of intelligence functions without depending upon language skills, and is therefore appropriate for the assessment of cognitive abilities of ethnic minorities and/or children with problems with verbal communication. The two subsets were mosaics, which assesses spatial visualization abilities and categories, which assesses abstract reasoning abilities (correlation with complete test *r* = 0.86). Raw test scores were converted into nonverbal IQ scores using normal values tailored to exact age. Thyroid dysfunction was defined as having a (standardized or absolute) TSH and/or FT4 below the 2.5th percentile or above the 97.5th percentile, defined in TPOAb negative women.

### Statistical analyses

We standardized (e.g., de-trended or residualized) TSH and FT4 concentrations to gestational age (or hCG concentrations in parallel analyses) continuously utilizing the Generalized Additive Models for Location Scale and Shape (GAMLSS). These specific statistical tools enable continuous, flexible, (semi) parametric normal score calculations while accounting for skewness and kurtosis of the data during the modeling process. We used three restricted cubic splines for gestational age at blood sampling or hCG, and linear gestational age at blood sampling or hCG for sigma variation and a Box Cox *t* family distribution (after sensitivity analyses using Akaike Information Criterion and worm plots) in order to achieve the best fit (29). Subsequently, gestational age-specific Z-scores were derived from the model, resulting in TSH and FT4 Z-scores which are interpretable relative to the gestational age, much like the widely used pediatric growth charts are interpretable relative to age. In parallel, the same methodology was used to create hCG-specific Z-scores.

First, to investigate the clinical characteristics of standardization of TSH and FT4 to gestational age at blood sampling (or hCG in parallel), we used multivariable linear regression models to study i) the association of gestational age at blood sampling with TSH and FT4 and ii) the association of standardized TSH and FT4 with non-standardized TSH and FT4. Next, we assessed the proportion of women reclassified with abnormal TSH and FT4 values, defined as the 2.5th and 97.5th percentiles of the population-based reference interval in TPOAb-negative women, according to non-standardized and gestational age standardized values. Finally, we used multivariable linear regression models to study the association of standardized to gestational age and non-standardized TSH and FT4 with offspring birth weight and IQ. The association of standardized and non-standardized TSH and FT4 with premature delivery and pre-eclampsia was investigated using multivariable logistic regression models.

For all analyses, we assessed potential non-linearity in linear and logistic regression models using restricted cubic splines utilizing three to five knots. For covariates with missing data (ranging from 0% for child sex and maternal age to <3.5% for TSH, FT4 or hCG, 7.1% for maternal education and 13.0% for maternal smoking), imputation according to the Markov Chain Monte Carlo method was used. All analyses were adjusted for relevant covariates including maternal age, BMI, ethnicity, education level, smoking and fetal sex, which were gathered through measurements during an intake visit, postal questionnaires, midwives and hospital registries (20, 21, 30).

## Results

**Figure 1:**
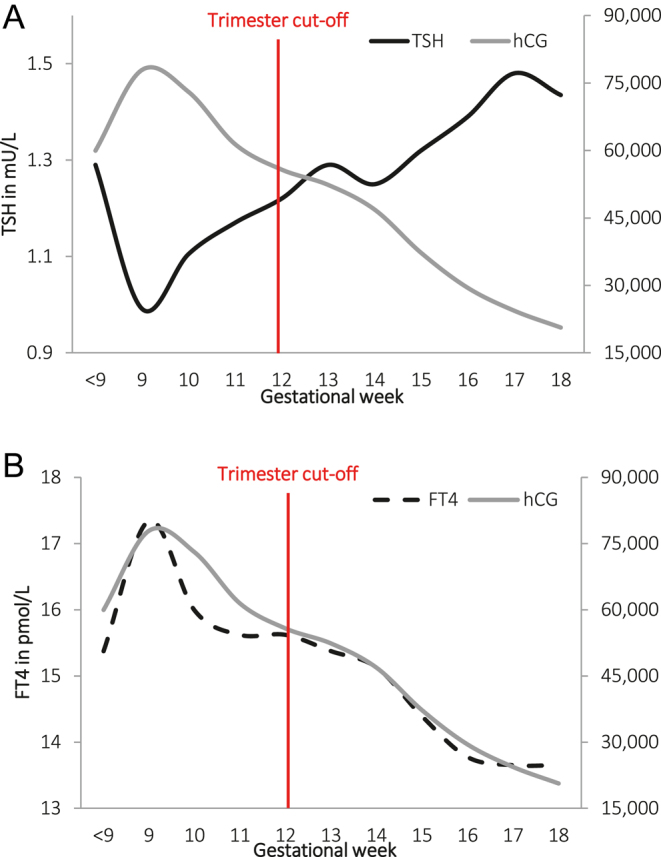
Course of hCG, TSH and FT4 during early pregnancy in TPOAb-negative women. The course of median hCG, TSH and FT4 during early pregnancy. The data are shown as the median levels per gestational week after exclusion of TPOAb-positive women.

**Figure 2:**
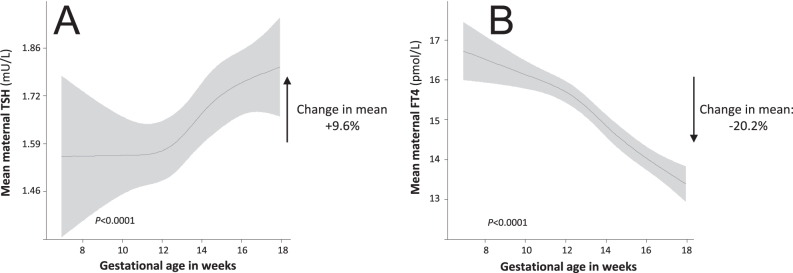
The association of gestational age with maternal TSH and FT4 levels.

A visualization of the concept of adjusting for gestational age for the interpretation of thyroid function tests, illustrated using FT4, is included as the Supplemental Fig. 1 (see the section on Supplementary materials given at the end of the article). Of the 7,069 women that were enrolled during early pregnancy (<18 weeks), TSH, FT4, TPO antibodies (TPOAbs) or hCG concentrations and gestational age at blood sampling were available in a total of 5,968 women. After exclusion of women with twin pregnancies (*n* = 128), pre-existing thyroid disease or thyroid-interfering medication usage (*n* = 89) and women with fertility treatment (*n* = 76), the final study population included 5,675 women. Descriptive statistics of the final study population and the course of thyroid function tests during pregnancy are shown in Fig. 1 and Supplemental Table 1, respectively. There was a positive association of gestational age at blood sampling with TSH, with a difference in the mean TSH between the 8th and 18th week of pregnancy of +9.6% (Fig. 2A). There was a negative association of gestational age at blood sampling with FT4, with a difference in the mean FT4 between the 8th and 18th week of pregnancy of −20.2% (Fig. 2B).

**Figure 3:**
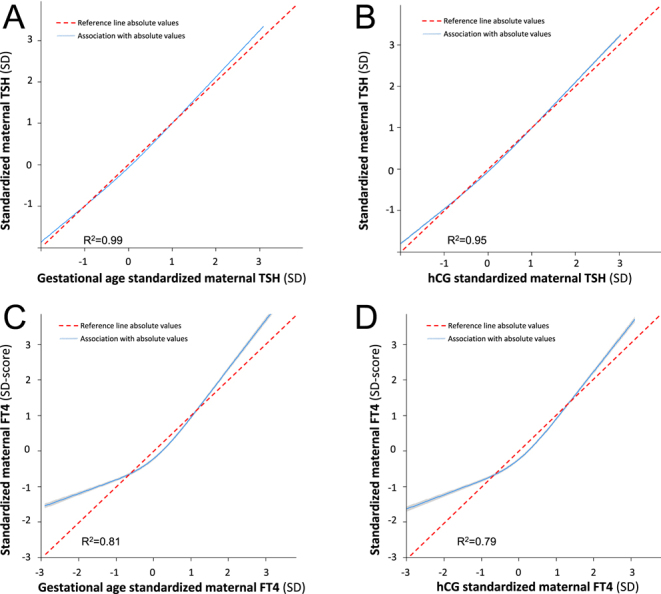
The differences between absolute and standardized maternal TSH and FT4 levels.

For TSH, there was a high correlation of absolute TSH values, with TSH values standardized to gestational age (variance of TSH explained by standardized TSH (*R*^2^) 99%; Fig. 3A) or standardized to hCG (*R*^2^ 95%; Fig. 3B). For FT4 either in the lower range or the higher range, those that were standardized to gestational age were relatively higher than the non-standardized values (Fig. 3C). A similar discrepancy was identified for FT4 values when standardized to hCG (Fig. 3D). FT4 standardized to gestational age or hCG explained, respectively, 81 and 79% of the variance in non-standardized FT4. In addition, TSH and FT4 concentrations standardized to gestational age were highly similar with those standardized to hCG (explained variance of 96 and 93% for TSH and FT4, respectively).

### Reclassification of abnormal TSH or FT4

Subsequently, we quantified the reclassification of abnormal TSH or FT4 after the standardization of TSH and FT4 to gestational age. We assessed the number of women that had an abnormal TSH (<2.5th percentile or >97.5th percentile regardless of gestational age) without standardization but a normal TSH after standardization to gestational age, and *vice versa* (Table 1). With the use of standardized TSH, 9.9% of all women with low (11%) or high (9.2%) TSH were now considered to have a normal TSH (Table 1). Conversely, of the women that were considered to have a normal TSH, 0.5% were now considered to have an abnormal TSH (0.2% reclassified to low TSH and 0.3% reclassified to high TSH; Table 1). With the use of standardized FT4, 20.3% of all women with low (23%) or high FT4 (17.7%) were now considered to have normal FT4. Conversely, of the women that were considered to have a normal FT4, 1.1% were now considered to have an abnormal FT4 (0.8% reclassified to low FT4 and 0.2% reclassified to high FT4; Table 1).

**Table 1:** Reclassification of abnormal TSH or FT4 according to non-standardized (NS) to gestational age standardized (STD) values. Data are presented as the number (%) of participants.

Cut-off	NS	STD	Reclassified to		
			Normal	Low	High
TSH					
Low	145	142	16 (11%)		
Normal	5,312	5,321		13 (0.2%)	14 (0.3%)
High	218	212	20 (9.2%)		
Total abnormal (% of total)	363 (6.4)	354 (6.2)			
FT4					
Low	148	159	34 (23%)		
Normal	5,369	5,374		45 (0.8%)	12 (0.2%)
High	158	142	28 (17.7%)		
Total abnormal (% of total)	306 (5.4)	301 (5.3)			

Low and high cut-off values were determined by the 2.5th and 97.5th percentile of a population-based reference interval calculated after the exclusion of TPOAb-positive women.

### Differences in the risk of adverse clinical outcomes

Next, we assessed if gestational age standardization of TSH and FT4 would change the association of abnormal TSH or FT4 with adverse clinical outcomes. Abnormal TSH or FT4 was defined according to non-standardized or gestational age standardized concentrations, and the risk with various adverse outcomes was compared. The change in effect estimates for the association of TSH with pre-eclampsia, stratified per quintile, varied from +3.2% to −10.6% with the use of gestational age standardized TSH (Table 2). The change in effect estimates for the association of high-normal FT4 with pre-eclampsia varied from −38.9% to −70.9% with the use of gestational age standardized FT4 (Table 2).

**Table 2:** The association of standardized (STD) and non-standardized TSH and FT4 with the risk of pre-eclampsia.

	Non-standardized		STD to gestational age		Effect estimate difference
	aOR* (95% CI)	*P*	aOR (95% CI)	*P*	
TSH					
1st quintile	1.61 (0.91–2.86)	0.103	1.44 (0.81–2.53)	0.213	−10.6%
2nd quintile	0.62 (0.31–1.25)	0.179	0.64 (0.33–1.27)	0.205	+3.2%
3rd quintile (ref)					
4th quintile	1.21 (0.67–2.18)	0.530	1.14 (0.66–1.97)	0.655	−5.8%
5th quintile	1.00 (0.55–1.84)	0.992	0.90 (0.49–1.65)	0.733	−10.0%
FT4					
1st quintile	1.69 (0.85–3.33)	0.134	0.99 (0.57–1.73)	0.976	−41.4%
2nd quintile	1.68 (0.85–3.30)	0.135	0.70 (0.38–1.30)	0.258	−58.3%
3rd quintile (ref)					
4th quintile	1.79 (0.90–3.60)	0.101	0.52 (0.27–1.01)	0.061	−70.9%
5th quintile	2.08 (1.05–4.12)	0.035	1.27 (0.97–1.68)	0.384	−38.9%

* All models were adjusted for relevant confounders, except gestational age when indicated.

aOR, adjusted odds ratio; CI, confidence interval.

Gestational age standardization had no considerable effects on the association of high TSH with premature delivery (aOR (95% CI): 1.75 (1.05–2.94) versus 1.77 (1.04–3.01); +1.1%; Table 3). The effect estimates of the association of high TSH with very premature delivery attenuated by 32.2% when high TSH was defined according to gestational age standardized TSH (aOR: 2.24 (0.95–5.29) versus 1.54 (0.55–4.31); Table 3). When low FT4 was defined according to FT4 standardized to gestational age, the effect estimates for the association with premature delivery attenuated by 6.3% (aOR: 2.39 (1.37–4.15) versus 2.24 (1.30–3.88)) and for very premature delivery these were attenuated by 16.1% (aOR: 4.04 (1.83–8.93) versus 3.39 (1.49–7.73); Table 3).

**Table 3:** The association of standardized and non-standardized TSH and FT4 with the risk of prematurity.

	Premature delivery (<37 weeks)			Very premature delivery (<34 weeks)		
	aOR* (95%CI)	*P*	EED	aOR (95%CI)	*P*	EED
High TSH (>97.5th percentile)			+1.1%			−31.2%
Non-standardized	1.75 (1.05–2.94)	0.033		2.24 (0.95–5.29)	0.047	
Standardized to gestational age	1.77 (1.04–3.01)	0.035		1.54 (0.55–4.31)	0.408	
Low FT4 (<2.5th percentile)			−6.3%			−16.1%
Non-standardized	2.39 (1.37–4.15)	0.002		4.04 (1.83–8.93)	0.001	
Standardized to gestational age	2.24 (1.30–3.88)	0.004		3.39 (1.49–7.73)	0.004	

* All models were adjusted for relevant confounders, except gestational age when indicated.

aOR, adjusted odds ratio; CI, confidence interval; EED, effect estimate difference.

Gestational age standardization had no considerable effects on the association of FT4 with birth weight (Supplemental Fig. 2B and C; corresponding β values: −0.04, −0.05 for statistical adjustment (reference) and gestational age standardization, respectively). Gestational age standardization also had no considerable effects on the association of FT4 with offspring IQ (Supplemental Fig. 3B and C; corresponding *β* values: 1.5 and −2.0; 1.5 and −2.0 for statistical adjustment and gestational age standardization, respectively).

There were no considerable differences in the effect estimates for the association of thyroid function with any outcome between any statistical adjustment or statistical adjustment for gestational age (Supplemental Figs 2A and 3A, Supplemental Table 2AB).

## Discussion

In the current study, we investigate the relevance of taking gestational age in to account when interpreting TSH and FT4 values by comparing the differences between non-standardized TSH and FT4 versus fully gestational age standardized TSH and FT4 and the association with the most relevant thyroid-related adverse pregnancy outcomes. The main finding of this study is that standardization of TSH and FT4 to gestational age at blood sampling weakens the effect estimates of the association of TSH and FT4 with adverse pregnancy outcomes (pre-eclampsia and premature delivery), but does not affect any associations with child outcomes (birth weight and IQ). These findings suggest that gestational age standardization, either by trimester or week-specific reference intervals, may not improve the identification of women at a higher risk of adverse pregnancy outcomes in early pregnancy.

During early pregnancy, FT4 and TSH concentrations change considerably due to varying concentrations of thyrotropic hCG (1, 25). These changes occur not only across trimesters but also within a single trimester (1, 5), leading to wide reference intervals. International guidelines recommend the use of trimester-specific reference intervals for TSH and FT4 to account for these changes (3, 4), although concrete evidence is lacking whether this approach better predicts adverse outcomes compared to non-pregnancy reference intervals or other methods. Since major changes in thyroid hormones peak at the end of the first and the beginning of the second trimester, coinciding with hCG peaks and the highest FT4 and lowest TSH levels, trimester-specific reference intervals may primarily identify women at these stages. This overlap between trimesters likely results in wide intervals because of the limited sensitivity in capturing the full spectrum of physiological changes, which is highlighted by the trend of TSH and FT4 in Fig. 1. Previous research has indeed found differences to be small between first and second trimester reference intervals, especially for TSH (14). In the current study, standardizing TSH and FT4 concentrations to gestational age at blood sampling weakened the association between thyroid function and adverse pregnancy outcomes, with no notable effects on child outcomes. This suggests that standardization of TSH or FT4 to gestational age, including the division in trimesters, offers no benefit in the 8th to 18th week of pregnancy (the inclusion period for this study). Instead, other determinants of thyroid hormones might prove to be superior with regard to defining different cohorts of gestational thyroid dysfunction, for instance, with the use of polygenic risk scores (31) or BMI categories (32). The results of the current study could also be regarded as an argument against the use of trimester-specific TSH or FT4 reference intervals during the first half of pregnancy, raising important considerations with regard to the discussion on screening for gestational thyroid dysfunction. Since screening test accuracy, reliability and reproducibility are considered vital criteria before commencing any screening program (33), it is imperative to review and potentially improve current recommendations.

Interestingly, large differences exist between studies that report on the association of gestational thyroid function with pregnancy or child outcomes with respect to the extent of gestational age standardization (34, 35). While most studies statistically adjust for gestational age, some studies calculated trimester-specific reference intervals (36, 37), some used standardized scores per week (38, 39, 40) or per increase in gestational age continuously (41, 42), and some do not standardize (43, 44). In the current study, we show that standardizing TSH and FT4 concentrations can lead to a decrease in the effect estimates of up to 71%. Additional studies are needed to investigate the role of gestational age standardization during very early (<8th week) and later (>18 weeks) pregnancy. To clarify, we used gestational age standardization in this study to evaluate the concept of standardization, acknowledging this is not a feasible approach to be implemented in clinical practice. There are many impracticalities for the incorporation into clinical practice, which also apply for week-specific or trimester-specific reference ranges, albeit to a different extent. We believe that future recommendations should recommend the simplest form of all reference interval determinations, for instance, the use of (adjusted) non-pregnancy reference intervals that still adequately identifies women at risk of adverse pregnancy outcomes related to gestational thyroid disease.

The lower effect estimates for pregnancy outcomes but not for child outcomes in this study could have various explanations. First, standardization to gestational age is a better approximation for the expected area under the curve for TSH or FT4 concentrations of the mother throughout the whole pregnancy, which could be described as the differential normalization for different periods of pregnancy. This could lead to differences between outcomes if certain outcomes are more strongly associated with thyroid hormone availability during very early pregnancy (such as placentation), or the first half of pregnancy, such as fetal brain development (45). The attenuation of the effect estimates for pre-eclampsia and prematurity might be explained by a better approximation of the thyroid hormone availability during very early pregnancy when using standardized values. Both pre-eclampsia (27) and prematurity (46) were previously associated with thyroid hormones, of which the effect was found to be mediated by hCG, a marker of placentation. Another explanation could be that the biological mechanisms by which thyroid hormone availability can affect adverse pregnancy outcomes may differ between pregnancy outcomes and child outcomes. Organogenesis and neurodevelopment go through multiple phases during pregnancy, and it is likely that different stages of development are affected differently by thyroid hormone availability, which could contribute to these discrepant results (47). Adverse effects may also be related to absolute extreme values of thyroid hormones, which peak during the end of the first trimester. Since full standardization to gestational age results in equal consideration of relatively high values at every time point, an association with absolute high values may be diluted. Finally, at least part of our results can be explained by the concept that standardization of TSH and FT4 to gestational age at blood sampling may have various (unwanted) effects; standardization changes the variance and the units of the measurement, which change the interpretation of the measurement and may over-standardize TSH and/or FT4 to factors that are associated with gestational age.

We were able to study gestational age-related changes in maternal TSH and FT4 concentrations and the effects of gestational age adjustment and standardization in the association of TSH and FT4 with several adverse outcomes in a large population-based sample using detailed phenotype data including the most relevant thyroid-related adverse pregnancy outcomes. Furthermore, the availability of hCG enabled us to study the effects of adjustment to gestational age at blood measurement from a more biological point of view and also add a form of internal replication to our findings. Our study was limited by the fact that TSH and FT4 were measured once during early pregnancy. This makes our results less generalizable to women that are <8 or >18 weeks pregnant. However, most women first attend a pregnancy clinic during the gestational age within which our study was performed and the changes in TSH and FT4 concentrations during pregnancy are most relevant during this period around the peak of hCG concentrations. The absence of repeated thyroid function measurements also limited us to study within-person variability, which could also be a relevant factor when it comes to the risk of adverse outcomes, especially since thyroid function abnormalities often normalize during gestation (48). Although gestational changes as assessed by between-subject differences of TSH, FT4 and hCG in a large population show very similar trends as compared to longitudinal studies (49, 50), future studies utilizing repeated measurement will be needed to further clarify the potential mechanisms underlying the current results.

In conclusion, standardizing TSH and FT4 reference intervals to gestational age between weeks 8–18 of pregnancy does not improve the identification of women at risk of pregnancy-related adverse outcomes previously related to gestational thyroid disease. As a consequence, the usefulness of trimester-specific reference intervals should be questioned, especially given that changes in thyroid physiology peak during the transition of the first to the second trimester. Future studies are needed to replicate our results and investigate whether there could be a benefit of using gestational age-specific reference intervals before the 8th or after the 18th week of pregnancy.

## Supplementary materials



## Declaration of interest

TIMK received lectureship fees from Merck, Berlin Chemie and IBSA. LC received research support in-kind by Abbott diabetes care division. The other authors have nothing to disclose.

## Funding

This study is funded by a Vidi grant (016.176.331) from the Netherlands Organization for Scientific Research (RPP), by a clinical fellowship from ZonMw, project number 90700412 (RPP) and by the ATHENA project, funded under the European Union's Horizon 2020 Program for research, technological development and demonstration, grant no. 825161.

## Author contribution statement

JO, TK, RP, and HT helped with investigation, methodology, and project administration. Formal analysis, software, visualization, and writing of the original draft were handled by JO and TK. LC, SvdB, VJ, ES, HT, and RP helped in data curation, methodology, resources, writing of the review and editing. Conceptualization and supervision were done by TK and RP. Funding acquisition was done by TK, VJ, ES, HT, and RP.
