# Supplementary material for: Standardization of TSH and FT4 to gestational age in early pregnancy and associations with clinical outcomes
Source: Eur Thyroid J. 2025 Jul 22;14(4):e240344. doi: 10.1530/ETJ-24-0344 (PMC12284872; doi:10.1530/ETJ-24-0344)
Supplement: Supplementary file 1 [file supplementary_materials.pdf]

Supplemental figure 1 Gestational age adjustment according to different strategies

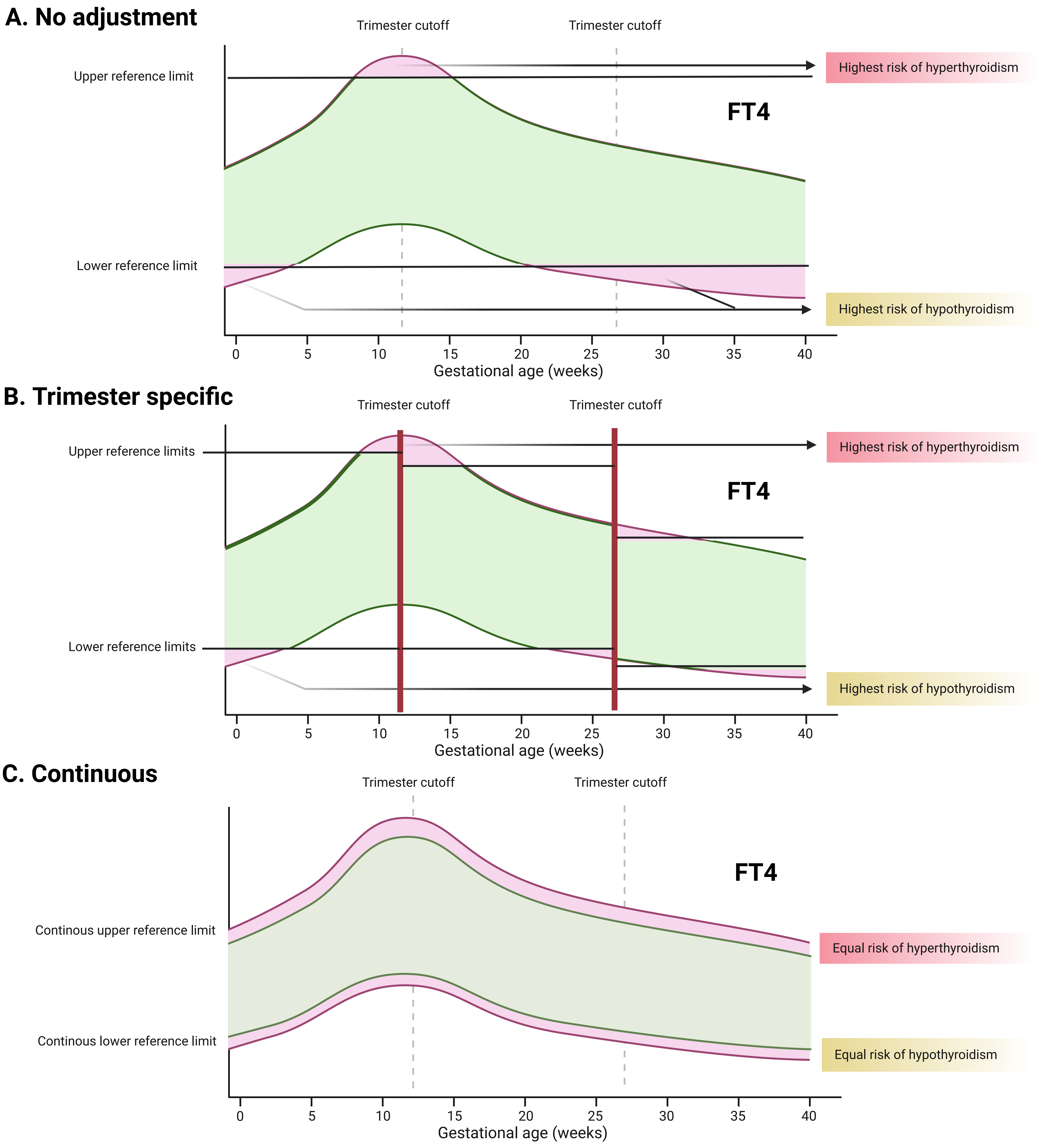

Supplemental Figure 2 Association of maternal FT4 with birthweight

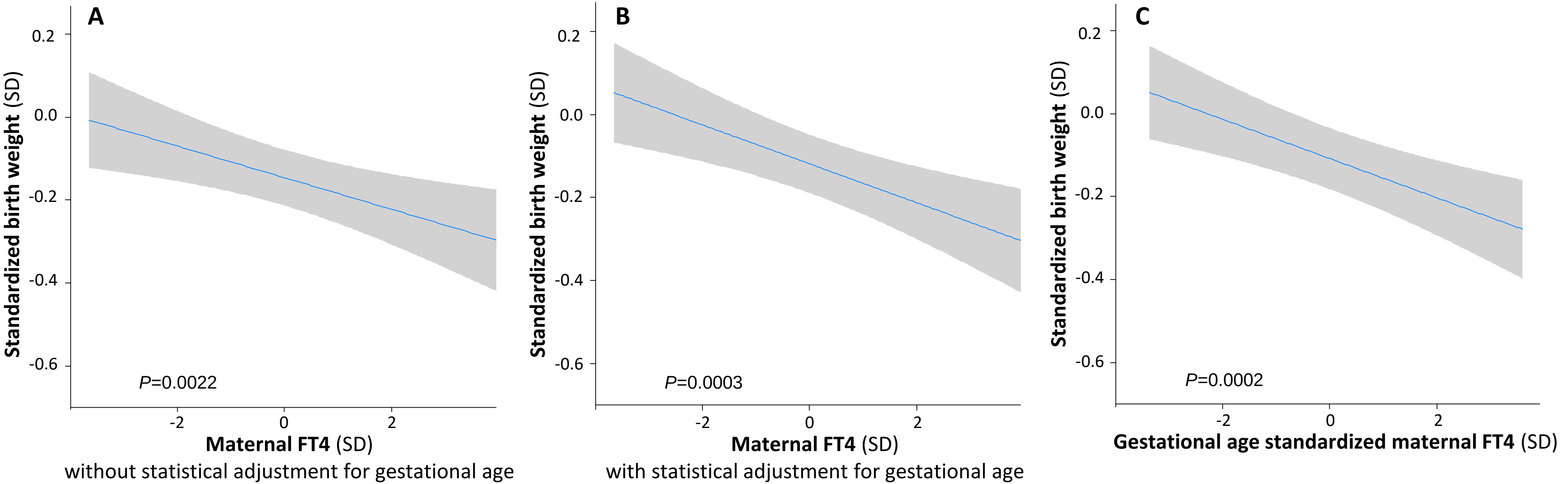

Supplemental Figure 3 Association of maternal FT4 with child IQ

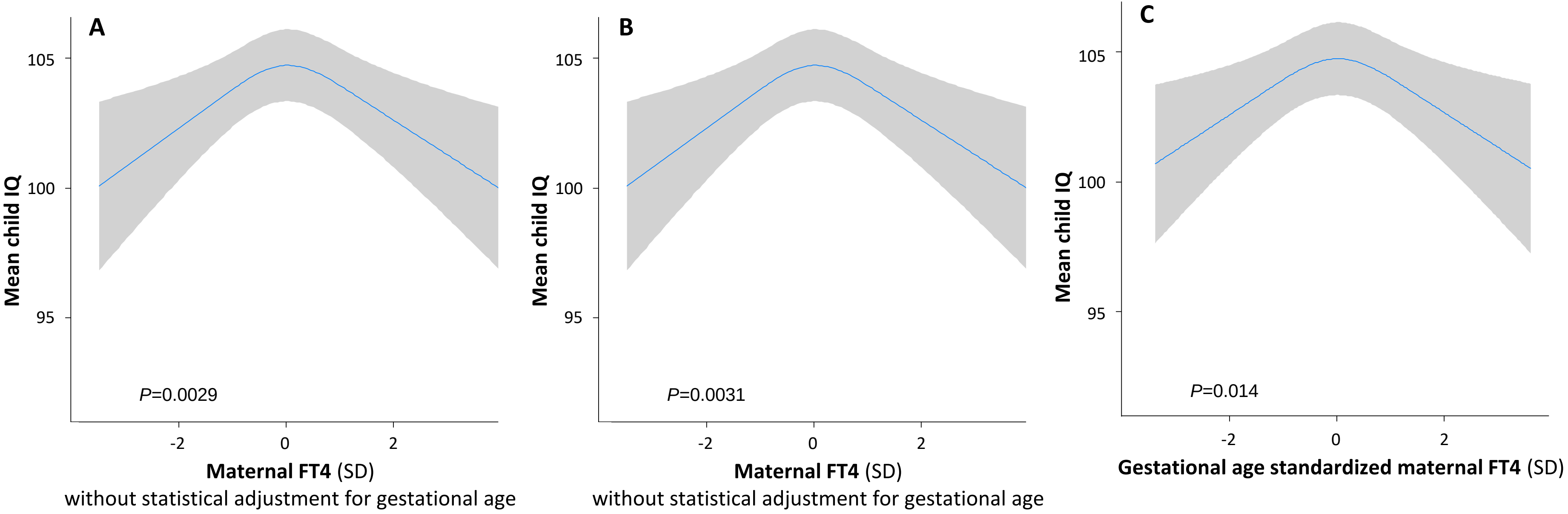

Supplemental Table 1 Descriptive statistics of final study population

**Population characteristics**	
N	5675
**Maternal characteristics**	
Age (years)	29.7 (5.1)
Gestational age (weeks)	13.2 (4.5-17.9)
BMI (kg/m2)	23.5 (4.2)
Parity, N (%)	
0	3228 (57.4%)
1	1675 (29.8%)
2	520 (9.2%)
≥3	156 (2.8%)
Active smoking	
Non/past smoker	3631 (73.5%)
Current smoker	450 (9.1%)
**Maternal test results**	
TSH (mU/L)	1.3 (0-4.5)
FT4 (pmol/L)	14.8 (10.3-22.3)
TPOAb positivity, N (%)	298 (5.3%)
**Pregnancy and Child characteristics**	
Preeclampsia	139 (2.7%)
Gestational age at birth (weeks)	39.9 (1.9)
Prematurity (<37 weeks)	286 (5%)
Birthweight (grams)	3413 (563.9)
Mean IQ (SD)	101.5 (14.9)

Data is presented as mean (standard deviation) or as median (range) as appropriate.

Supplemental Table 2a The association of standardized and non-standardized TSH and FT4 with the risk of pre-eclampsia.

	aOR* (95%CI)	*P*	aOR (95%CI)	*P*
**TSH**	**Non-adjusted for gestational age**		**Adjusted for gestational age**	
1st quintile	1.56 (0.88-2.77)	0.127	1.61 (0.91-2.86)	0.103
2nd quintile	0.61 (0.30-1.24)	0.174	0.62 (0.31-1.25)	0.179
3rd quintile (ref)	ref		ref	
4th quintile	1.21 (0.67-2.18)	0.520	1.21 (0.67-2.18)	0.530
5th quintile	1.00 (0.54-1.83)	0.990	1.00 (0.55-1.84)	0.992

	aOR (95%CI)	*P*	aOR (95%CI)	*P*
**FT4**	**Non-adjusted for gestational age**		**Adjusted for gestational age**	
1st quintile	1.77 (0.90-3.49)	0.100	1.69 (0.85-3.33)	0.134
2nd quintile	1.71 (0.87-3.36)	0.122	1.68 (0.85-3.30)	0.135
3rd quintile (ref)	ref		ref	
4th quintile	1.74 (0.87-3.49)	0.121	1.79 (0.90-3.60)	0.101
5th quintile	1.96 (1.00-3.87)	0.051	2.08 (1.05-4.12)	0.035

*All models were adjusted for relevant confounders, except gestational age when indicated. aOR: Adjusted Odds Ratio, CI: Confidence interval

Supplemental Table 2b The association of TSH and FT4 with the risk of prematurity with and without statistical adjustment for gestational age.

	**Premature delivery** (<37 weeks)		**Very premature delivery** (<34 weeks)	
	aOR* (95%CI)	*P*	aOR (95%CI)	*P*
**TSH**				
Non-adjusted for gestational age	1.75 (1.05-2.94)	0.033	2.24 (0.95-5.29)	0.064
Adjusted for gestational age	1.75 (1.05-2.94)	0.033	2.24 (0.95-5.29)	0.047
**FT4**				
Non-adjusted for gestational age	2.41 (1.39-4.18)	0.002	4.15 (1.89-9.09)	<0.001
Adjusted for gestational age	2.39 (1.37-4.15)	0.002	4.04 (1.83-8.93)	0.001

*All models were adjusted for relevant confounders, except gestational age when indicated. aOR: Adjusted Odds Ratio, CI: Confidence interval
